# Tannery Effluent Treatment by Yeast Species Isolates from Watermelon

**DOI:** 10.3390/toxics5010006

**Published:** 2017-02-04

**Authors:** Stanley Irobekhian Reuben Okoduwa, Bernard Igiri, Chinyere Blessing Udeh, Chidi Edenta, Balli Gauje

**Affiliations:** 1 Directorate of Research and Development, Nigerian Institute of Leather and Science Technology, Zaria 810221, Nigeria; egwubernard2@gmail.com; 2 Infohealth Awareness Division, SIRONigeria Global Limited, Abuja 900288, Nigeria; cudeh56@yahoo.com; 3 Department of Biochemistry, Renaissance University, Ugbawka, Enugu State 402211, Nigeria; chidiedenta@gmail.com; 4 Environmental Technology Division, National Research Institute for Chemical Technology, Zaria 810282, Nigeria; write2bally@gmail.com

**Keywords:** effluent, environmental risk, health-hazards, *Saccharomyces cerevisiae*, *Torulaspora delbrueckii*

## Abstract

The quest for an effective alternative means for effluent treatment is a major concern of the modern-day scientist. Fungi have been attracting a growing interest for the biological treatment of industrial wastewater. In this study, *Saccharomycescerevisiae* and *Torulasporadelbrueckii* were isolated from spoiled watermelon and inoculated into different concentrations of effluent. The inoculants were incubated for 21-days to monitor the performance of the isolates by measurement of biochemical oxygen demand (BOD), chemical oxygen demand (COD), nitrates, conductivity, phosphates, sulphates and turbidity. The results showed that *Saccharomycescerevisiae* had the highest percentage decrease of 98.1%, 83.0%, 60.7%, 60.5%, and 54.2% for turbidity, sulphates, BOD, phosphates and COD, respectively, of the tannery effluent. *Torulasporadelbrueckii* showed the highest percentage decrease of 92.9%, 90.6%, and 61.9% for sulphates, COD, and phosphates, respectively, while the syndicate showed the highest percentage reduction of 87.4% and 70.2% for nitrate and total dissolve solid (TDS), respectively. The least percentage decrease was displayed by syndicate organisms at 51.2%, 48.1% and 40.3% for BOD, COD and conductivity, respectively. The study revealed that *Saccharomycescerevisiae* and *Torulasporadelbrueckii* could be used in the biological treatment of tannery-effluent. Hence, it was concluded that the use of these organisms could contribute to minimizing the adverse environmental risks and health-hazards associated with the disposal of untreated tannery-effluents.

## 1. Introduction

Generally, in developing countries such as Nigeria, the vast majority of industries dispose their effluents without treatment. The discharge of tannery effluent, municipal sewage, farm and urban wastes carried by drains to rivers worsens and broadens water pollution [1]. These effluents have dangerous consequences on the quality of water and the environment, as well as multifaceted effects on flowing water [2]. The tannery industry generates massive amounts of effluents during its regular operation. These effluents contain tannins, significant concentrations of biochemical oxygen demand (BOD) and chemical oxygen demand (COD), and inorganic compounds such as sulphates, chlorides and some toxic heavy metals which are difficult to treat and therefore are released untreated or partially treated into the environment [3,4,5]. Tannery effluents on the surface of water bodies leach into ground water which leads to contamination with toxic metallic components such as: Cr^6+^, Fe^3+^ [6], Cd (ll) [7], Cu(ll) [8]. Tannery effluents contain several pollutants, including Cr (III), NaS, NaCl, and the aftermath of the discharge of these effluents can cause a serious problem for living organisms [9]. For instance, the high value of COD and BOD in the soil leads to the depletion of dissolved oxygen in the soil which has an adverse effect on aquatic life and also inhibits the life of soil microorganisms [10,11]. Effluent treatment would help prevent the contamination of drinking water and contaminants entering into the food chain [12]. Many traditional methods used for tannery wastewater treatment include coagulation/flocculation, electrochemical treatment [13,14,15], the activated sludge process, the sequential batch reactor, and the up flow anaerobic sludge blanket [16,17]. The physical methods, chemical methods or a combination of both are more effective for the treatment of tannery effluents because the chemical degradation rate is faster than biological degradation rate [14,15]. The tannery industry was chosen in this study because it generates large amounts of untreated effluents containing large amounts of chemicals into the environment. The biological techniques for the treatment of tannery effluents, such as biosorption or bioaccumulation strategies, may provide attractive alternatives to existing technologies, such as the electrochemical method, which are very expensive [18,19]. Both living (bioaccumulation) and non-living (biosorption) microbial biomass can act as effective metal scavengers [20,21]. Watermelon undergoing spoilage contains microbes (yeast species) that can be used to remediate polluted environments containing organic and chemical substances in industrial effluents. The choice of isolating microbes from spoiled watermelon was made to recycle the watermelon, which is seen as waste in our society, in order to clean the environment. The advantage of *Saccharomyce cerevisiae* and *Torulaspora delbrueckii* isolates obtained from spoiled watermelon over other microbes in the treatment of tannery effluent is their ability to reduce inorganic compounds, such as sulphides, ammonia and total nitrogen, rapidly. The removal or reduction of harmful substances from tannery effluents by microbe-based technologies may provide an alternative or additional means of waste recovery for economic reasons and environmental protection. The aim of this research is to use yeast (*Saccharomyce cerevisiae* and *Torulaspora delbrueckii*) isolates obtained from spoiled watermelon for the treatment of tannery effluents.

## 2. Materials and Methods

### 2.1. Collection of the Samples

Composite effluents (combination of soaking effluent, unhairing/liming effluent and tanning effluent) was collected using a clean plastic can from the point of discharge into the environment at the Nigerian Institute of Leather and Science Technology, (NILEST) Zaria, Kaduna State, Nigeria. The effluent sample collected was transferred to the laboratory in ice-cold container and immediately kept at 4 °C until analysis.

Samples of spoiled watermelons were collected from Nyanya Market, Abuja, Nigeria. The spoiled watermelons were collected using a clean polyethylene bag and transferred to the Microbiology Laboratory at BioRapid Diagnostic Laboratory Research Center, Abuja, Nigeria where it was kept in the refrigerator until analysis.

### 2.2. Isolation of Yeast

The isolation of yeast was done using Saboroud Dextrose Agar (SDA) by the pour plate method. The fleshy part of the spoiled watermelon, about 2 g was inoculated into test tube containing 8.0 mL of sterile water. It was thereafter diluted serially to 10^−8^ dilution factor. As described by American Public Health Association [22]. Exactly 0.5 mL of the serially diluted samples (10^−7^ and 10^−8^) was inoculated into a petri dish and 25 mL of SDA was pour, smear and incubated at 25 °C for 48 h [22]. Thereafter, two different yeast of distinct colony were isolated, sub-cultured separately on SDA and incubated at 25 °C for 24 h. A stock culture was made for each of the yeast species by means of slant bottle containing SDA and labeled *Saccharomyces cerevisiae* and *Torulaspora delbrueckii* after characterization. 2.5 mL each from the stock suspension of *Saccharomyces cerevisiae* and *Torulasporadel brueckii* were mixed to form the syndicate organisms.

### 2.3. Characterization of the Yeast Isolates

These were done based on the sugar fermentation method [22]. In brief, a loop full of the test organisms (*Saccharomyces cerevisiae* and *Torulaspora delbrueckii*) each was inoculated separately into a fermentation medium containing fructose, glucose, maltose, mannose, lactose, sorbitol and sucrose using phenol red as indicator. Durham tube was introduced into the test tube containing fructose, glucose, maltose, mannose, lactose, sorbitol and sucrose and incubated at 25 °C for 24 h. A change in color of the medium from red to yellow indicated acid production while, a displacement at the top of the inverted Durham tube indicated gas production.

### 2.4. Experimental Procedure for Effluent Treatment

The procedure described by APHA [22] was used. A loop full of the *Saccharomyces cerevisiae* and *Torulaspora delbrueckii* from each of the stock culture was inoculated into Potato Dextrose Broth (PDB) and incubated for 48 h at 25 °C. After which, 5 mL of each yeast culture was inoculated to the different concentrations of the effluent [(100 mL of effluent) as 100% effluent, (75 mL of effluent plus 25 mL of tap water) as 75% effluent and (50 mL of effluent plus 50 mL of tap water) as 50% effluent]. These were contained in different 250 mL conical flask and labelled as (*Saccharomyces cerevisiae* and effluent, *Torulaspora delbrueckii* and effluent, *Saccharomyces cerevisiae*, *Torulaspora delbrueckii* and effluent). A control flask without yeast cells was also set up. They were kept in an orbit shaker for three weeks and maintained at 27 ± 2 °C. The experiments were set up in triplicate and at 100%, 75% and 50% concentrations of the effluent.

### 2.5. Analysis of Physicochemical Properties

The physicochemical properties of the effluent were carried out before treatment and consecutively on weekly bases as a means of monitoring the pollution. Saratale et al. [23] and APHA [24] methods for effluent treatment were adopted for all the physicochemical properties of the effluent that analyzed. Dissolve oxygen (DO) was determined using DO meter (Lutron DO-5519, Taipei, Taiwan).

#### 2.5.1. Nitrate

First 25 mL of the effluent was dispensed into a reduction column followed by addition of 75 mL NH_4_Cl-EDTA solution and mixed. The mixed sample was collected at the rate of 10 mL·min^−1^ into original sample flask. After reduction, 2.0 mL color reagent was added to 50 mL sample and mixed. The resulting solution was allowed between 10 min and 2 h. The absorbance was then measured at 543 nm against a distilled water reagent blank using spectrophotometer (HACH DR 2400 portable spectrophotometer, Loveland, CO, USA).

#### 2.5.2. Phosphate

First 50 mL of the effluent was dispensed into 125 mL Erlenmeyer’s flask and one drop of phenolphthalein indicator was added. A drop of 5 N H_2_SO_4_ solution was added to discharge the color. Then 8 mL combined reagent was added, mixed thoroughly and allowed for 10 min. The absorbance was then measured at 880 nm, using reagent blank as the reference solution.

#### 2.5.3. Sulphate

Accurately, 100 mL of the effluent was dispensed into 250 mL Erlenmeyer’s flask and 0.3 g of BaCl_2_ crystals was added and stirred for 1 min. After stirring, the sample was placed in 5 cm cuvette for 4 min and the absorbance was measured at 420 nm.

#### 2.5.4. Chemical Oxygen Demand (COD)

Dichromate reactor digestion methods were used, small volumes of the effluent sample (2 cm^3^) was pipette into vials containing the premeasured reagents, including catalysts and chloride compensator. The vial was incubated at 150 °C for 2 h for digestion to take place and allowed to cool. The COD measurement was carried out using HACH 890/DR Colorimeter.

#### 2.5.5. Turbidity

Turbidity was measured in Nephelometric Turbidity Units (NTU). The turbidity meter was ON, and cleaned prepared calibration standards cal 800 NTU were inserted and read. The first one was removed and the second one cal 200 NTU was inserted and read. After the second one was read and removed, the third one cal 100 NTU was inserted and read. The sample cell was filled with the sample and inserted into sample holder. Enter key was pressed and the turbidity value was read and recorded.

#### 2.5.6. Biochemical Oxygen Demand (BOD)

Initial dissolved oxygen (DO) was determined using DO meter. Incubation of bottle containing desired sample (90 mL) was done at 20 ± 1 °C. The final DO was determined with DO meter (Lutron DO-5519, Taipei, Taiwan).

(1) $$\mathrm{Calculation}:\;\mathrm{BOD}\left(\mathrm{mg}\cdot L^{-1}\right)=\frac{D1-D2}{P}$$

where:

*D*1 = DO of diluted sample immediately after preparation (mg·LG^−1^)
*D*2 = DO of diluted sample after 5 days incubation at 20 °C (mg·LG^−1^)
*P* = Decimal volumetric fraction of sample used (mL of the sample taken (90 mL) divided by total volume of the BOD bottle (250 mL).

### 2.6. Statistical Analysis

Values of results obtained was expressed as mean ± standard deviation. Percentage change was calculated between the controls and experimental for the various experiments.

## 3. Results and Discussion

The results of the analysis of the degradation of tannery effluents using *Saccharomyces cerevisiae* and *Torulaspora delbrueckii* isolated from spoiled watermelon for a period of 21 days are presented in Table 1, Table 2 and Table 3. The initial physicochemical properties at different concentrations (100%, 75% and 50%) were observed to be very significantly high and the reduction rate by *Saccharomyces cerevisiae*, *Torulaspora delbrueckii* and the syndicate was rapid at the initial time of the treatment, with a sharp reduction in the parameters at day 7. This is due to the removal of the organic load and toxicity from the tannery effluent by the yeast ant it is in agreement with the findings reported by Ong et al. [25] and Abioye et al. [26]. However, at day 14, some physicochemical properties such as the BOD, COD, and conductivity increased in *Saccharomyces cerevisiae*–treated effluent (Table 1). This may be due to the death of some organisms and/or saturation of the organisms’ binding site. This observation was in accordance with the findings reported by Abioye et al. [26]. In *Torulaspora delbrueckii*– and syndicate-treated effluent, there was a continuous reduction of the various parameters measured at day 14 and 21, except for conductivity and sulphates which increased at day 14 in *Torulaspora delbrueckii*, with continuous reduction in syndicate organisms. This could be attributed to the efficiency of both the living and death cells and the beneficial synergy of the syndicate organisms.

**Figure 1:**
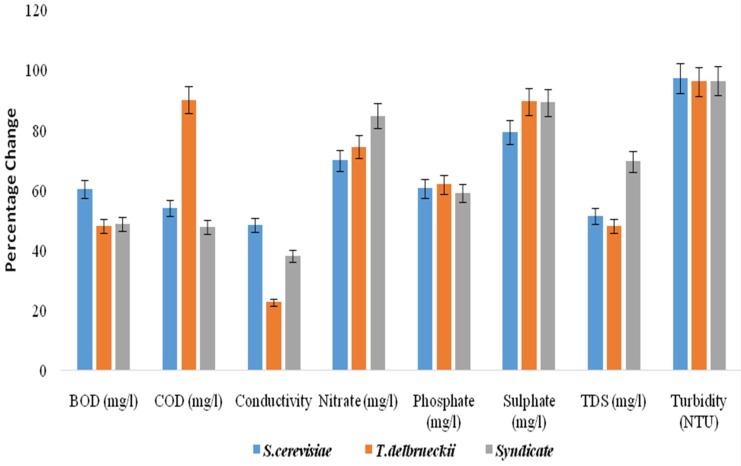
Percentage change in physicochemical parameters after treatment of 50% tannery effluent with individual yeast species and syndicate.

**Figure 2:**
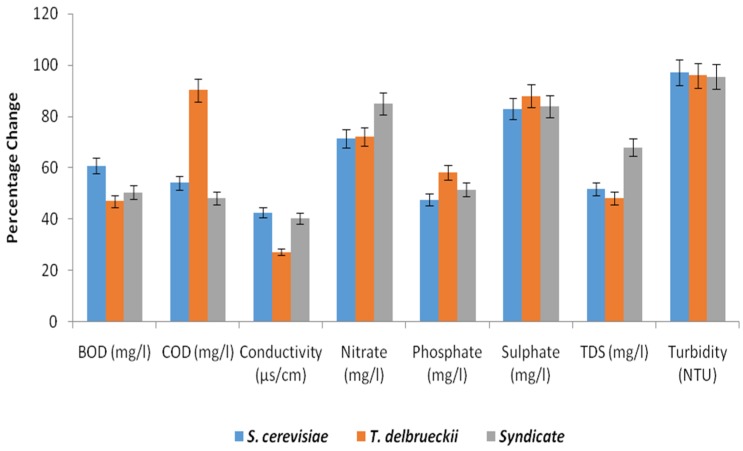
Percentage change in physicochemical parameters after treatment of 75% tannery effluent with individual yeast species and syndicate.

**Figure 3:**
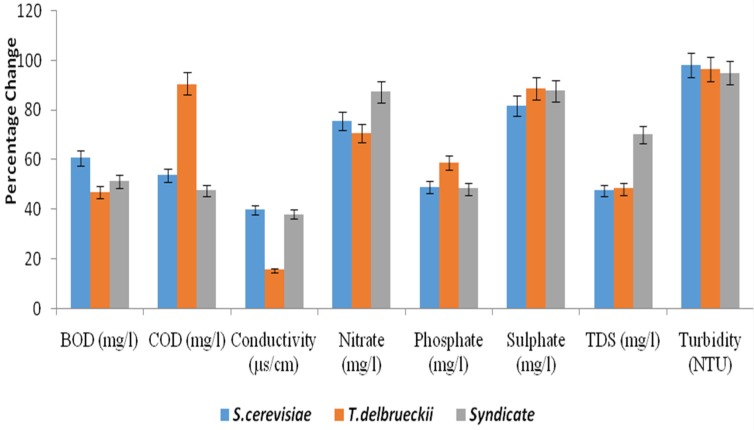
Percentage change in physicochemical parameters after treatment of 100% tannery effluent with yeast species and syndicate.

The mean values of the BOD of the effluents before treatment at different concentrations (100%, 75% and 50%) were 4248 ± 6.52 mg/L, 4237 ± 5.20 mg/L and 4267 ± 11.03 mg/L, which were beyond the permissible limit (30 mg/L) of the Central Pollution Control Board [20] for disposal. However, after degradation, Saccharomyces cerevisiae degraded the BOD to 1671 ± 3.65 mg/L, 1664 ± 2.75 mg/L and 1693 ± 5.07 mg/L (Table 1), *Torulasporadelbrueckii* degraded the BOD to 2250 ± 9.50 mg/L, 2244 ± 9.89 mg/L and 2217 ± 11.90 mg/L (Table 2), and the syndicate organisms degraded the BOD to 2075 ± 7.89 mg/L, 2097 ± 4.78 mg/L and 2188 ± 7.98 mg/L (Table 3) and their percentage changes were), 48.0% (Figure 1), and 51.2% (Figure 2) and 60.7% (Figure 3).

The mean values of the COD of the effluent before treatment at different concentrations (100%, 75% and 50%) were 10,607 ± 11.02 mg/L, 10,656 ± 9.42 mg/L and 10,593 ± 7.04 mg/L which were beyond the permissible limit (250 mg/L) [27] for the disposal of effluent. However, after the degradation, *Saccharomyces cerevisiae* degraded the COD to 4910 ± 7.15 mg/L, 4879 ± 9.14 mg/L and 4863 ± 11.03 mg/L (Table 1). *Torulaspora delbrueckii* degraded the COD to 997 ± 6.98 mg/L, 1032 ± 9.89 mg/L and 1045 ± 5.79 mg/L (Table 2), and the syndicate organisms degraded the COD to 5560 ± 4.78 mg/L, 5533 ± 2.65 mg/L and 5547 ± 6.87 mg/L (Table 3) and their percentage changes were 54.20% (Figure 2), 90.60% (Figure 3), and 48.10% (Figure 2). These findings were in agreement with the work of Noorjaham [28] which reported a mean value of 2637 ± 2.32 mg/L before treatment and 297 ± 1.58 mg/L after treatment with a percentage change of 88.73%.

The TDS mean values of the effluent before treatment at different concentrations (100%, 75% and 50%) were 248 ± 11.23 mg/L, 265 ± 3.56 mg/L and 253 ± 4.90 mg/L, which were below the permissible limit (2100 mg/L) approved by the Central Pollution Control Board [20] for the disposal of effluents. Nevertheless, after degradation, *Saccharomyces cerevisiae* degraded the TDS to 130 ± 6.15 mg/L, 128 ± 2.98 mg/L, and 123 ± 6.87 mg/L, *Torulaspora delbrueckii* degraded the TDS to 128.3 ± 7.90 mg/L, 137.2 ± 7.39 mg/L and 131 ± 5.90 mg/L, and the syndicate organisms degraded the TDS to 74 ± 4.78 mg/L, 85 ± 6.89 mg/L and 77 ± 1.45 mg/L and their percentage changes were 51.7%, 48.3%, and 70.2%, respectively. This was in disagreement with the work of Noorjaham [21] which reported a mean value of 6428 ± 3.3115 mg/L before treatment, 2045 ± 1.5811 mg/L after treatment and a percentage change of 53.54%.

The phosphates and TDS of *Saccharomyces cerevisiae*–, *Torulaspora delbrueckii*– and syndicate organisms – treated tannery effluent at different concentrations fell within the acceptable limits allowed for waste water discharge by the Central Pollution Control Board (CPCB) [4] and the World Health Organization standard. The values were 1.68–1.96 mg/L and 123–130 mg/L, respectively, with a percentage reduction of 60.5%, and 51.7% for *Saccharomyces cerevisiae* (Table 1), 1.47–1.62 mg/L and 128.3–137.2 mg/L, respectively, with a percentage reduction of 61.9% and 48.3% for *Torulaspora delbrueckii* (Table 2) and 1.74–1.84 mg/L and 74–85 mg/L, with a percentage reduction of 59.1% and 70.2% for syndicates organisms (Table 3).

The turbidity, nitrates and sulphates of the effluent after 21 days treatment were in the range of 15.9–18.3 NTU, 65.3–76.8 mg·L^−1^ and 11.6–13.4 mg·L^−1^, respectively, with percentage decreases of 96.6%, 74.5% and 89.5%, and the values fell within the permissible limit of the Central Pollution Control Board [20].

To select the yeast isolate with higher bioremediation potential out of the two species isolated from the spoiled watermelon, the percentage reduction potentials produced in different parameters were compared. It was observed that there was a higher percentage reduction of turbidity (98.1%), COD (90.60%), sulphates (89.5%), nitrates (87.4%) and TDS (70.2%) when the tannery effluent was inoculated with *Saccharomyces cerevisiae*, *Torulaspora delbrueckii* and syndicates organisms.

## 4. Conclusions

From the results obtained at the end of the study, it was observed that *Saccharomyces cerevisiae* and *Torulaspora delbrueckii* isolated from spoiled watermelon have the potential to degrade the physicochemical parameters present in the raw tannery wastewater to a relatively low level in favorable conditions. When comparing the levels of reduction in the pollution strength for the samples containing the microbes, the yeast species (*Saccharomyces cerevisiae* and *Torulaspora delbrueckii*) met at least some of the standards set by the Environmental Protection Agency (EPA) of the United State of America and the Central Pollution Control Board (CPCB) of New Delhi. Hence, spoiled watermelon can be used as a source of raw material for the biological treatment of tannery effluents.

## Figures and Tables

**Table 1:** The physicochemical features of different concentrations of tannery effluents treated with *Saccharomyces cerevisiae*.

Parameters	Conc. of Effluents	Post Treatment Days			
		0	7	14	21
BOD (mg/L)	100%	4249 ± 6.52	2990 ± 7.24	3142 ± 11.10	1671 ± 3.65
COD (mg/L)		10,607 ± 11.02	7475 ± 9.25	7863 ± 7.06	4910 ± 7.15
Conductivity (µs/cm)		412 ± 5.25	515 ± 11.24	527 ± 4.51	248 ± 12.71
Nitrates (mg/L)		262 ± 4.53	105 ± 9.72	60 ± 4.64	64 ± 9.27
Phosphates (mg/L)		3.56 ± 7.12	3.48 ± 9.34	2.74 ± 5.24	1.82 ± 6.52
Sulphates(mg/L)		115 ± 6.42	35 ± 2.78	27 ± 6.83	21 ± 9.11
TDS (mg/L)		248 ± 11.23	173 ± 4.67	130 ± 5.45	130 ± 6.15
Turbidity (NTU)		474 ± 4.67	27 ± 7.86	20 ± 9.04	9 ± 5.81
BOD (mg/L)	75%	4237 ± 5.20	2969 ± 6.03	3176 ± 5.50	1664 ± 2.75
COD (mg/L)		10,656 ± 9.42	7485 ± 7.06	7940 ± 6.05	4879 ± 9.14
Conductivity (µs/cm)		378 ± 7.05	521 ± 9.64	521 ± 6.47	217 ± 11.42
Nitrates (mg/L)		249 ± 6.72	97 ± 6.72	65 ± 7.50	71 ± 5.67
Phosphates (mg/L)		3.74 ± 9.67	3.62 ± 4.23	3.23 ± 6.81	1.96 ± 4.78
Sulphates(mg/L)		112 ± 6.81	32 ± 4.93	26 ± 5.92	19 ± 9.56
TDS (mg/L)		265 ± 3.56	169 ± 6.86	136 ± 7.85	128 ± 2.98
Turbidity (NTU)		463 ± 8.23	24 ± 6.97	17 ± 4.98	13 ± 7.34
BOD (mg/L)	50%	4267 ± 11.03	2964 ± 9.05	3137 ± 7.06	1693 ± 5.07
COD (mg/L)		10,593 ± 7.04	7439 ± 6.05	7926 ± 7.15	4863 ± 11.03
Conductivity (μs/cm)		399 ± 9.24	503 ± 7.71	519 ± 7.60	206 ± 9.53
Nitrates (mg/L)		256 ± 7.62	111 ± 7.54	72 ± 8.04	77 ± 6.65
Phosphates (mg/L)		4.25 ± 7.98	3.17 ± 6.87	2.68 ± 2.98	1.68 ± 6.12
Sulphates(mg/L)		111 ± 4.67	34 ± 4.98	24 ± 5.70	23 ± 7.09
TDS (mg/L)		253 ± 4.90	181 ± 8.09	142 ± 11.15	123 ± 6.87
Turbidity (NTU)		447 ± 6.98	21 ± 5.87	19 ± 11.03	12 ± 5.89

**Table 2:** The physicochemical features of different concentrations of tannery effluents treated with *Torulaspora delbrueckii*.

Parameters	Conc. of Effluents	Post Treatment Days			
		0	7	14	21
BOD (mg/L)	100%	4249 ± 6.52	2950 ± 7.89	2836 ± 4.78	2250 ± 9.50
COD (mg/L)		10,607 ± 11.02	7395 ± 6.98	7083 ± 4.98	997 ± 6.98
Conductivity (µs/cm)		412 ± 7.90	541 ± 5.98	585 ± 7.12	476 ± 4.56
Nitrates (mg/L)		262 ± 5.87	196 ± 7.98	143 ± 5.87	76.8 ± 9.23
Phosphates (mg/L)		3.56 ± 6.34	3.16 ± 7.98	2.17 ± 6.78	1.47 ± 9.67
Sulphates (mg/L)		115 ± 11.12	9.6 ± 11.45	18.9 ± 8.13	12.9 ± 6.89
TDS (mg/L)		248 ± 11.23	211.8 ± 6.89	169.4 ± 4.87	128.3 ± 7.90
Turbidity (NTU)		474 ± 6.45	88.3 ± 7.11	27.2 ± 5.98	15.9 ± 6.09
BOD (mg/L)	75%	4237 ± 5.20	2958 ± 5.78	2828 ± 7.90	2244 ± 9.89
COD (mg/L)		10,656 ± 9.42	7363 ± 8.90	7049 ± 5.89	1032 ± 9.80
Conductivity (µs/cm)		378 ± 6.89	535 ± 4.78	582 ± 7.23	481 ± 8.90
Nitrates (mg/L)		249 ± 4.89	187 ± 9.23	138 ± 5.23	69.8 ± 9.04
Phosphates (mg/L)		3.74 ± 2.98	3.13 ± 6.75	2.11 ± 3.98	1.56 ± 7.09
Sulphates (mg/L)		112 ± 5.56	7.9 ± 4.97	21.7 ± 7.45	13.4 ± 8.34
TDS (mg/L)		265 ± 3.56	203.5 ± 7.34	178.3 ± 4.89	137.2 ± 7.39
Turbidity (NTU)		463 ± 3.89	98.5 ± 5.67	23.8 ± 6.87	18.3 ± 5.89
BOD (mg/L)	50%	4267 ± 11.03	2981 ± 6.89	2815 ± 7.45	2217 ± 11.90
COD (mg/L)		10,593 ± 7.04	7406 ± 2.34	7070 ± 4.87	1045 ± 5.79
Conductivity (µs/cm)		399 ± 9.86	524 ± 8.56	572 ± 4.89	489 ± 9.87
Nitrates (mg/L)		256 ± 4.97	176 ± 6.98	127 ± 4.23	65.3 ± 2.12
Phosphates (mg/L)		4.25 ± 5.66	3.24 ± 5.78	2.09 ± 6.77	1.62 ± 11.02
Sulphates (mg/L)		111 ± 2.45	8.4 ± 7.32	14.9 ± 8.10	11.6 ± 7.34
TDS (mg/L)		253 ± 4.90	205.7 ± 4.85	183.7 ± 5.85	131.6 ± 5.90
Turbidity (NTU)		447 ± 4.21	76.4 ± 6.89	20.3 ± 6.45	16.8 ± 7.34

**Table 3:** The physicochemical features of different concentrations of tannery effluents treated with the syndicate.

Parameters	Conc. of Effluents	Post Treatment Days			
		0	7	14	21
BOD (mg/L)	100%	4249 ± 6.52	3229 ± 5.87	2635 ± 6.98	2075 ± 7.89
COD (mg/L)		10,607 ± 11.02	7805 ± 5.56	6315 ± 3.56	5560 ± 4.78
Conductivity (µs/cm)		665 ± 5.78	579 ± 11.45	479 ± 6.89	412 ± 3.78
Nitrates (mg/L)		262 ± 6.89	115 ± 4.67	78 ± 4.67	33 ± 5.34
Phosphates (mg/L)		3.56 ± 4.98	2.88 ± 9.22	2.06 ± 5.98	1.84 ± 6.87
Sulphates (mg/L)		115 ± 2.67	40 ± 6.87	16 ± 2.45	14 ± 8.13
TDS (mg/L)		248 ± 11.23	176 ± 5.87	101 ± 5.11	74 ± 4.78
Turbidity (NTU)		474 ± 2.11	67 ± 5.89	37 ± 3.67	23 ± 5.87
BOD (mg/L)	75%	4237 ± 5.20	3243 ± 1.34	2614 ± 5.89	2097 ± 4.78
COD (mg/L)		10,656 ± 9.42	7791 ± 5.87	6309 ± 6.88	5533 ± 2.65
Conductivity (µs/cm)		633 ± 7.89	568 ± 5.98	493 ± 3.33	378 ± 4.90
Nitrates (mg/L)		249 ± 3.78	107 ± 3.78	96 ± 2.54	37 ± 11.90
Phosphates (mg/L)		3.74 ± 4.89	2.81 ± 2.11	2.15 ± 7.90	1.81 ± 9.09
Sulphates (mg/L)		112 ± 3.09	26 ± 5.98	22 ± 5.98	18 ± 5.23
TDS (mg/L)		265 ± 3.56	169 ± 5.98	110 ± 4.87	85 ± 6.89
Turbidity (NTU)		463 ± 2.56	55 ± 3.76	43 ± 3.23	21 ± 4.78
BOD (mg/L)	50%	4267 ± 11.03	3243 ± 4.90	2625 ± 9.89	2188 ± 7.98
COD (mg/L)		10,593 ± 7.04	7780 ± 4.89	6322 ± 2.78	5547 ± 6.87
Conductivity (µs/cm)		643 ± 7.34	582 ± 3.89	476 ± 5.87	399 ± 2.23
Nitrates (mg/L)		256 ± 4.98	117 ± 4.98	81 ± 4.78	39 ± 3.98
Phosphates (mg/L)		4.25 ± 9.45	2.72 ± 6.34	2.17 ± 2.56	1.74 ± 4.78
Sulphates (mg/L)		111 ± 7.23	35 ± 2.11	21 ± 5.89	12 ± 4.45
TDS (mg/L)		253 ± 4.90	181 ± 4.34	98 ± 7.67	77 ± 1.45
Turbidity (NTU)		447 ± 6.78	48 ± 2.76	34 ± 4.44	17 ± 2.78
